# Recurrent glioblastoma in national guidelines on the diagnosis and treatment of gliomas: A matter of European practice variation

**DOI:** 10.1016/j.bas.2024.103923

**Published:** 2024-10-10

**Authors:** Mark P. van Opijnen, Rob J.A. Nabuurs, Filip Y.F. de Vos, Mohini T.R. Ramsoedh, Joost J.C. Verhoeff, Marjolein Geurts, Marike L.D. Broekman

**Affiliations:** a Department of Neurosurgery, Leiden University Medical Center, Albinusdreef 2, 2333 ZA, Leiden, the Netherlands; b Department of Cell and Chemical Biology, Leiden University Medical Center, Albinusdreef 2, 2333 ZA, Leiden, the Netherlands; c Department of Neurosurgery, Haaglanden Medical Center, Lijnbaan 32, 2512 VA, The Hague, the Netherlands; d Department of Neurosurgery, Haga Teaching Hospital, Els Borst-Eilersplein 275, 2545 AA, The Hague, the Netherlands; e Department of Medical Oncology, Utrecht University Medical Center, Utrecht, the Netherlands; f Department of Medicine, University of Leiden, Leiden, the Netherlands; g Department of Radiation Oncology, Amsterdam University Medical Center, Amsterdam, the Netherlands; h Brain Tumor Center at Erasmus MC Cancer Institute, University Medical Center Rotterdam, Rotterdam, the Netherlands

**Keywords:** Glioblastoma, Recurrence, Treatment, Guideline, Practice variation

## Abstract

**Introduction:**

The optimal treatment for recurrent glioblastoma patients remains not well-defined in international guidelines. On top of that, the availability of national guidelines is uncharted.

**Research question:**

This study aimed to investigate the availability of national guidelines on the diagnosis and treatment of adult glioma throughout Europe, specifically focusing on recurrent glioblastoma.

**Material and methods:**

Medical specialists with neuro-oncology expertise from all European countries were asked for the availability of official national guidelines. The primary outcome was whether guidelines provided recommendations on the treatment of recurrent glioblastoma in adults. Secondary outcomes included treatment specific recommendations and the role of clinical trials in the treatment of recurrent glioblastoma. The quality of the guidelines was assessed using the AGREE II instrument.

**Results:**

Of the 50 countries in Europe, information on guideline availability was obtained for 38 countries (76%). In twelve countries (24%) national guidelines on the diagnosis and treatment of glioma in adults exist. Focusing on recurrent glioblastoma, nine (18%) of the European countries provided any recommendations on the treatment of recurrent glioblastoma. In four (33%) guidelines it was explicitly stressed that there is currently no standard or evidence-based treatment for these patients.

**Discussion and conclusion:**

National guidelines on the treatment of glioblastoma in adults are not uniformly available in Europe. In addition, and in contrast with international guidelines, the national guidelines differ profoundly in their recommendations regarding recurrent glioblastoma. This could contribute to unwanted practice variation. Efforts are needed to not only optimize, but also harmonize treatment for recurrent glioblastoma patients.

## Highlights

• National guidelines on adult glioma are widely unavailable in Europe.
• Nearly a fifth of the countries provide recommendations on recurrent glioblastoma.
• Recommendations on recurrent glioblastoma differ profoundly between countries.
• Quality of guidelines vary remarkably between domains and between countries.
• International agreement on treatment of recurrent glioblastoma is needed.

## 1 Introduction

The optimal diagnosis and treatment for primary glioblastomas in adults is well-defined in (inter)national guidelines. For instance, the guideline of the European Association for Neuro-Oncology (EANO) on the diagnosis and treatment of diffuse gliomas of adulthood provides clear, evidence-based recommendations for the treatment of newly diagnosed *IDH*-wild-type glioblastoma (Weller et al., 2021). However, in the recurrent setting, an inevitable and dismal scenario, evidence on the best treatment strategy becomes scarce and highly relies on individual patient characteristics. As the EANO guideline states, ‘standard-of-care treatments for patients with recurrent glioblastoma are not well-defined.’ (Weller et al., 2021) The only comment on recurrent glioblastoma in the guideline of the European Society for Medical Oncology (ESMO) is the lack of efficacy of erlotinib and imatinib (Stupp et al., 2014). The guideline of the American Society of Clinical Oncology (ASCO) and Society for Neuro-Oncology (SNO) is in line with the European tendency: ‘no recommendation for or against any therapeutic strategy can be made for treatment of recurrent glioblastoma.’ (Mohile et al., 2022).

Previously, we have shown that practice variation regarding recurrent glioblastoma re-resection even exists within one country (van Opijnen et al., 2023). This observation raised the question to what extent national guidelines in Europe, if any, provide recommendations on the treatment of recurrent glioblastoma. To help physicians and their patients find the best treatment option for recurrent glioblastoma, in the absence of clear international recommendations, national guidelines could play a role. National guidelines are of particular interest since availability of (experimental) therapies may vary *per* country as the implementation of current scientific evidence may differ.

This study aims to investigate the availability of national guidelines on the diagnosis and treatment of adult glioma throughout Europe, specifically focusing on recurrent glioblastoma. Since optimal treatment in the recurrent phase of the disease is not well-defined in international recommendations, we want to explore whether recommendations on the treatment of adult patients with recurrent glioblastoma are provided on a national level.

## 2 Material and methods

### 2.1 Study design

Medical specialists from all European countries were asked by email or in person for the availability of national guidelines in their country on the diagnosis and treatment of gliomas in adults. These medical specialists were neurosurgeons, neurologists with neuro-oncology expertise, medical oncologists or radiation oncologists, all involved in the care for patients with brain tumors in their country. When available, the document of what they currently use as a guideline was shared with us or could be downloaded directly from the Internet. Additional online mining was performed to retrieve information from the countries of which the contacted persons did not respond to our messages. Informal or incomplete documents such as letters, patient information folders, expert opinions or presentations were not included in this guideline study. For all other, official guidelines or consensus documents, the latest version available was used. Non-English and non-Dutch documents were carefully translated using online translation tools and were subsequently read. Various synonyms of ‘recurrent’ (e.g. ‘regrowth’, ‘relapse’, ‘recurrence’) were used to retrieve all information about recurrent glioblastoma or recurrent glioma in general.

### 2.2 Outcomes

The primary outcome was whether national guidelines provided recommendations on the treatment of recurrent glioblastoma in adults, regardless treatment modality or specific treatment details. This was divided into ‘No national guideline available’, ‘No recommendations’ and ‘Treatment recommendations’. Secondary outcomes included treatment specific recommendations and the role of clinical trials in the treatment of recurrent glioblastoma (divided into ‘No national guideline available’, ‘No recommendations’ and ‘Trial recommendations’).

### 2.3 Guideline quality assessment

The quality of the entire guidelines was assessed using the Appraisal of Guidelines for Research and Evaluation II (AGREE II) (Brouwers et al., 2010). The AGREE II is an internationally widely used and validated instrument for guideline appraisal (Hoffmann-Eßer et al., 2017). Using this instrument, six relevant domains, comprising 23 different items, were scored separately for each guideline. These domains were: scope and purpose, stakeholder involvement, rigour of development, clarity of presentation, applicability, and editorial independence. Each domain was scored using a scale ranging from 1 (strong disagreement) to 7 (strong agreement). This scoring was independently done by two appraisers (MPvO and MTRR) to improve quality assessment.

### 2.4 Statistical analysis

Categorical variables were reported by using counts and percentages while continuous variables were described by using the medians and ranges. Instructions in the user's manual of the AGREE II were followed to properly calculate the domain scores. For each domain, the maximum possible score was: *number of items per domain* x *highest possible score* (7) x *number of appraisers* (2). Likewise, the minimum possible score for each domain was: *number of items per domain* x *lowest possible score* (1) x *number of appraisers* (2). The following equation was used to scale the scores for each domain:

$$\frac{Obtained\;score-Minimum\;possible\;score}{Maximum\;possible\;score-Minimum\;possible\;score}\;x\;100\%$$

If an item was not included in the guideline, this absence of information was scored with a 1 out of 7. Total domain scores of ≥60% were deemed acceptable and scores ≥80% were deemed high quality (Barriocanal et al., 2016; Madera et al., 2018; Keaver et al., 2022). Figures were created using the open software environment *R*, version 4.2.1.

## 3 Results

### 3.1 General results

**Fig. 1:**
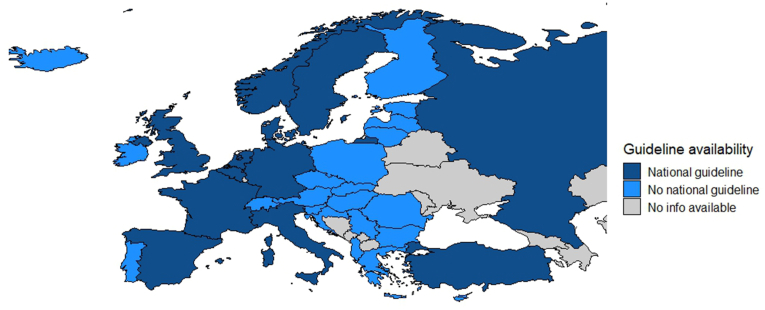
Guideline availability in Europe.

Of the 50 European countries (geographically defined and transcontinental countries included) (Countries-ofthe-world), information on the availability of national guidelines was obtained for 38 countries (76%). 26 (52%) of these countries did not and twelve (24%) did have a national guideline, respectively. The twelve countries from which their national guidelines on the diagnosis and treatment of gliomas in adults was shared were: Belgium, Denmark, France, Germany, Italy, the Netherlands, Norway, Russia, Spain, Sweden, Turkey and United Kingdom. The latest versions of the guidelines differed between 2008 and 2023 (median 2020). The guidelines either discussed neurological diseases in general (1/12), or neuro-oncological diseases (5/12), or gliomas (4/12), or glioblastomas specifically (2/12). See Fig. 1 for a visualization of the guideline availability.

### 3.2 Treatment recommendations

**Fig. 2:**
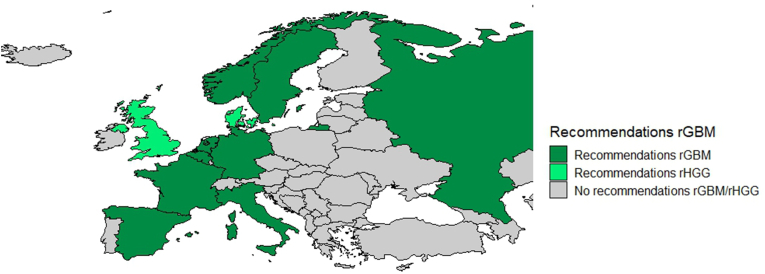
National recommendations on recurrent glioblastoma rGBM: recurrent glioblastoma; rHGG: recurrent high-grade glioma.

Of all 50 European countries, in nine (18%) national guidelines recommendations on the treatment of recurrent glioblastoma were provided – regardless of the comprehensiveness of the recommendations. The guidelines of two countries (Denmark and United Kingdom) reported only on recurrent high-grade glioma in general while the guideline of Turkey only reported on recurrent gliomas in general (Fig. 2).

For those twelve countries with national guidelines, in four of them (33%) it was explicitly stressed that there is currently no standard or evidence-based treatment for patients with recurrent glioblastoma. Multidisciplinary consultation to discuss treatment upon recurrence was recommended in seven (58%) guidelines, for the other countries it was not clear if this was so obvious that it was not stated, or that it was not common practice. As a time-dependent cut-off for treatment upon progression, a progression free period between initial tumor treatment and recurrence of at least six months was suggested in six (50%) of these guidelines. Palliative care and symptom management at first recurrence was suggested in all but the Turkish guideline (92%).

Re-resection as one of the treatment modalities was recommended for selected patients only in all available guidelines. Selection criteria for re-resection were generally the same across the countries, with Karnofsky performance status (KPS, e.g. ≥70), time to recurrence (e.g. more than six months) and the age of the patient (e.g. <70) as the most frequently mentioned prognostic factors to be taken into account. The specific cut-offs of these factors were not provided in all guidelines. The Belgian guideline stated that ‘selected patients with a focal recurrence’ might benefit from a second resection but did not further specify the selection criteria. Re-resection combined with the implementation of carmustine-impregnated wafers (Gliadel®) was considered as an option in the French and Spanish guidelines.

Although the majority of the guidelines mentioned the potential of either a rechallenge temozolomide after a temozolomide-free interval (e.g. of more than four to six months) or treatment with CCNU (lomustine), more variation was seen regarding other systemic treatment options. The anti-vascular endothelial growth factor (anti-VEGF) antibody bevacizumab, for instance, was suggested as an anti-tumor treatment option in the Danish, German and Russian guidelines. Regorafenib, an oral multi-targeted tyrosine kinase inhibitor, was considered in the Italian guideline as a first therapeutic option for patients with recurrent glioblastoma and with a good performance status (defined as KPS ≥80). The German guideline briefly referred to regorafenib, but none of the other guidelines mentioned this regimen. Likewise, dendritic cell based immunotherapy was suggested in a single guideline (Belgium). Treatment with tumor treating fields (TTFields) in case of recurrence was actively not recommended, as stated in the English, French, Italian, Norwegian, Spanish and Swedish guideline.

Regarding re-irradiation, there was a general consensus that only patients with a small, focal recurrence, and taken into account the previously administered dose and radiation-free interval (e.g. of six to twelve months), can be offered a second course of radiation therapy. Specific definitions of the factors to be taken into account were not provided in all guidelines.

### 3.3 Role of clinical trials

Regarding the role of clinical trials in the recurrent setting, five (42%) of the available guidelines considered enrollment into clinical trial to be an option. For example, the Spanish guideline stated that ‘the best option [for recurrent glioblastoma] is the enrollment into clinical trials’. If that is not an option, a second-line treatment should be considered according to this guideline. Genomic profiling in the context of enrollment into clinical trials was recommended in the Danish guideline. The guideline of the United Kingdom, however, stated that ‘the point at which to use genomic biomarker-based therapy’ is uncertain.

### 3.4 Quality assessment

#### 3.4.1 Scope and purpose

This domain is 'concerned with the overall aim of the guideline, the specific health questions, and the target population’ (Brouwers et al., 2010). The median score for this domain was 58.3% with a range of 13.9–100%. The Turkish guideline scored the lowest score. The English guideline had the highest score for this domain.

#### 3.4.2 Stakeholder involvement

This domain ‘focuses on the extent to which the guideline was developed by the appropriate stakeholders and represents the views of its intended users’ (Brouwers et al., 2010). The median score for this domain was 58.3% with a range of 0.0–86.1%. The French guideline scored the lowest score. The English guideline scored the highest score for this domain.

#### 3.4.3 Rigour of development

This domain ‘relates to the process used to gather and synthesize the evidence, the methods to formulate the recommendations, and to update them’ (Brouwers et al., 2010). The median score for this domain was 26.6% with a range of 13.5–79.2%. The French guideline scored had the lowest score. The Italian guideline scored highest for this domain.

#### 3.4.4 Clarity of presentation

This domain 'deals with the language, structure, and format of the guideline’ (Brouwers et al., 2010). The median score for this domain was 76.4% with a range of 44.4–97.2%. The Turkish guideline scored lowest. The Danish guideline scored highest for this domain.

#### 3.4.5 Applicability

This domain 'pertains to the likely barriers and facilitators to implementation, strategies to improve uptake, and resource implications of applying the guidelines’ (Brouwers et al., 2010). The median score for this domain was 20.8% with a range of 2.1–45.8%. The Belgian guideline had the lowest score. The English guideline scored highest.

#### 3.4.6 Editorial independence

This domain is 'concerned with the formulation of recommendations not being unduly biased with competing interests’ (Brouwers et al., 2010). The median score for this domain was 25.0% with a range of 0.0–79.2%. The French and Turkish guidelines scored lowest. The English guideline had the highest score for this domain.

#### 3.4.7 Overall assessment

According the AGREE II instrument manual, the abovementioned domain scores are independent and should not be aggregated into a single quality score (Brouwers et al., 2010). However, it is evident that the UK guideline had overall the highest scores (Table 1). Applicability of the different guidelines was generally low, while the clarity of presentation was generally good.

**Table 1:** Domain scores for the available guidelines using the AGREE-II instrument (Brouwers et al., 2010).

Country	Domain 1: scope and purpose (%)	Domain 2: stakeholder involvement (%)	Domain 3: rigour of development (%)	Domain 4: clarity of presentation (%)	Domain 5: applicability (%)	Domain 6: editorial independence (%)
Belgium	25.0%	41.7%	27.1%	61.1%	2.1%	29.2%
Denmark	58.3%	58.3%	28.1%	**97.2%**	33.3%	50.0%
France	19.4%	0.0%	13.5%	**86.1%**	14.6%	0.0%
Germany	77.8%	72.2%	49.0%	**88.9%**	12.5%	79.2%
Italy	**88.9%**	58.3%	79.2%	75.0%	31.1%	75.0%
The Netherlands	**97.2%**	66.7%	59.4%	75.0%	20.8%	20.8%
Norway	58.3%	58.3%	21.9%	75.0%	20.8%	33.3%
Russia	36.1%	38.9%	26.0%	50.0%	33.3%	20.8%
Spain	55.6%	50.0%	16.7%	77.8%	10.4%	20.8%
Sweden	69.4%	58.3%	21.9%	**86.1%**	35.4%	12.5%
Turkey	13.9%	38.9%	26.0%	44.4%	12.5%	0.0%
United Kingdom	**100%**	**86.1%**	74.0%	77.8%	45.8%	79.2%

Median score (range)	58.3% (13.9–100)	58.3% (0.0–86.1)	26.6% (13.5–79.2)	76.4% (44.4–97.2)	20.8% (2.1–45.8)	25.0% (0.0–79.2)

High quality scores (≥80%) depicted in bold, acceptable scores (≥60–79%) are underlined.

## 4 Discussion

This study investigated the availability of national guidelines on the diagnosis and treatment of adult glioma throughout Europe. Of the 50 European countries, twelve (24%) shared their national guidelines publicly online or through personal correspondence. Focusing on recurrent glioblastoma, we found that only nine (18%) of the European countries provide any recommendations on the treatment of recurrent glioblastoma. The quality of the twelve available guidelines assessed by the AGREE II method showed remarkable differences between countries and domains, with the guideline of the United Kingdom showing overall the highest scores.

Information on the treatment of recurrent glioblastoma varied from the statement that there is currently no standard-of-care for these patients, to more detailed descriptions of the (lack of) evidence for different treatment modalities. This was not explicitly taken into account in this study, since the first statement (i.e. ‘there is currently no standard-of-care') might as well provide guidance to clinicians. Moreover, the body of recurrent glioblastoma recommendations did not appear to be related to the quality of the guideline: some guidelines clearly provided different treatment options but showed marginal scores on the quality assessment, and vice versa. In general, this study did not intend to include sociodemographic characteristics or economic status to compare different guidelines and different countries, although it is not unlikely that this could affect the content of national recommendations.

Interestingly, the administration of bevacizumab as a treatment for glioblastoma recurrence was mentioned in some guidelines. However, this drug has only been approved for that indication outside the European Union, like in Canada, Switzerland and the United States, based on two uncontrolled phase 2 studies showing objective response rates of around 30% for the treatment with bevacizumab alone or in combination with irinotecan (Friedman et al., 2009; Kreisl et al., 2009). The European evidence-based opinion, however, is that there is no survival benefit of bevacizumab for the treatment of recurrent glioblastoma (Weller et al., 2021; Taal et al., 2014; Wick et al., 2010). Likewise, the application of carmustine wafers, as considered in two guidelines, is currently not common practice in Europe (Weller et al., 2021; Mohile et al., 2022).

Attention should be paid when presence of guidelines becomes synonymous to good clinical practice. As mentioned before, even in the presence of national guidelines remarkable differences in re-resection practice have been observed between neuro-oncology specialists (van Opijnen et al., 2023). Thus, national guidelines do not necessarily rule out the phenomenon of practice variation. Similarly, the absence of national guidelines does not necessarily mean suboptimal practice, especially when considering the availability of international guidelines. Indeed, some respondents stated that, in the absence of a national guideline, international guidelines (e.g. the EANO guideline) are used. Here, the adage ‘absence of evidence does not mean evidence of absence’ seems applicable. Nevertheless, the discrepancy in treatment uniformity between the primary setting and the recurrent setting, as observed in glioblastoma patients, remains worrisome. More importantly, prioritizing the collection of evidence in the recurrent setting should precede the development of guidelines, since the increasing number of guidelines is currently not paralleled by an equal increase in evidence. The development of more guidelines should therefore be viewed critically in the absence of more data and evidence on the treatment of recurrent glioblastoma.

The ASCO-SNO guideline strongly recommends the participation of recurrent glioblastoma patients in clinical trials were possible (Mohile et al., 2022). The EANO guideline agrees on this, albeit less pronounced, with the statement that appropriate clinical trials ‘should be considered' (Weller et al., 2021). However, only five (42%) of the available guidelines in our study considered enrollment into clinical trials as an option, with varying degrees of strength of that recommendation. Based on the lack of evidence for standard systemic treatment options and low availability for suited patients, we strongly advocate the enrollment of recurrent glioblastoma patients in clinical trials. As effective treatment options are still limited, identification of new clinically relevant targets is of urgent importance and should be done in the context of clinical trials and prospective registries (Capper et al., 2023; van Opijnen et al., 2022).

Some limitations of this study have to be considered when interpreting our findings. The design of the study potentially resulted in the retrieval of only those guidelines of countries with known or findable contact information. Details on the guideline availability of the twelve countries for which we have not been able to obtain any information would have been of added value. Second, the language in which the guidelines are written may have influenced proper interpretation, although careful reading and translation was pursued. Another limitation is the absence of country-specific clinical outcome data, that might have made the correlation possible between (presence of) national recommendations and clinical outcomes. Generally put, quantification of our data would be of interest.

## 5 Conclusion

In conclusion, this study shows that national guidelines on the treatment of recurrent glioblastoma in adults are widely unavailable in Europe. This, among other factors including education, patient volume, lack of evidence, and the role of multidisciplinary consultations, could contribute to unwanted (inter)national practice variation and should therefore force more (experimental) research into the optimal treatment for these patients. When comparing national guidelines, cultural and educational differences should be taken into account. Future research should investigate whether national guideline availability correlates with clinical outcomes and with sociodemographic characteristics and economic status of countries, in order to further study the impact and origins of unwanted (inter)national practice variation.

## Funding

None.

## Declaration of competing interest

None of the authors declare a conflict of interest.
